# Influence of Size and Maturity on Injury in Young Elite Soccer Players

**DOI:** 10.3390/ijerph18063120

**Published:** 2021-03-18

**Authors:** Natascia Rinaldo, Emanuela Gualdi-Russo, Luciana Zaccagni

**Affiliations:** Department of Neuroscience and Rehabilitation, Faculty of Medicine, Pharmacy and Prevention, University of Ferrara, 44121 Ferrara, Italy; rnlnsc@unife.it (N.R.); luciana.zaccagni@unife.it (L.Z.)

**Keywords:** youth athletes, soccer, injury, overuse, maturation, anthropometry

## Abstract

The involvement of pre-adolescents in soccer is becoming more and more frequent, and this growing participation generates some concerns about the potential factors for sports injuries. The purpose of this study was to investigate sports injuries in younger (U9–U11) and older (U12–U13) children playing soccer at an elite level, analyzing potential anthropometric and maturity risk factors. A total of 88 elite soccer players aged 9–13 years were investigated. Weight, stature, and sitting height were measured at the start and at the end of the competitive season, computing the relative growth velocities. Additional body composition parameters were taken during a second survey. Maturity offset was calculated using predictive equations based on anthropometric traits such as years from age at peak height velocity (YPHV). Injuries suffered during the competitive season were recorded. Maturity and some anthropometric characteristics were significantly different according to the presence or absence of injuries among the players. Multiple logistic regression revealed that YPHV, body mass index (BMI), and calf muscle area were the factors most significantly correlated with injuries. Players with increased BMI, with decreased calf muscle area, and who were closer to their peak height velocity, were at a higher risk of injury. Findings showed that a monitoring program of anthropometric characteristics taking into account the maturational stage needs to be developed to prevent injuries.

## 1. Introduction

Soccer is one of the most popular sports in the world, and it is especially enjoyed by children and teenagers. It is estimated that 3.9 million children and adolescents participate in soccer annually [1,2]. The psychological and physical health benefits of physical activity and sports practice are well known and documented [3,4,5,6]. However, given its nature as a high-intensity contact sport, soccer is associated with a great injury risk, which makes it a target sport for injury prevention [7,8].

For this reason, it is fundamental to analyze the factors related to injury occurrence in soccer players, especially in elite and sub-elite players, as they usually result in a considerable amount of time loss from training and matches and represent a significant economic burden for the health care system [1,9]. Previous studies analyzed the risk factors and the rate of injuries in adult elite soccer players, both males [7] and females [10], but there is a paucity of studies focusing on injury rate and modalities in children and adolescents [1,11]. Moreover, the studies focusing on this topic have several limitations [12] as they are usually based only on adolescents [9] or high school and college players [13]. Sometimes, they also report combined data from adolescents and adults [7], or they combine several sports [14].

Injury incidence among soccer players differs across levels of participation, age, type of exposure, and sex [1,8,15,16,17] with soccer players under the age of 12 years exhibiting a lower injury rate (1.0–1.6 injuries per 1000 h) compared to older players [1,16,17]. However, this rate varies considerably among different studies. It is estimated that 2,995,765 children and adolescents aged 7–17 had an emergency department visit in the United States during a 25-year epidemiological study, with an annual average of about 120,000 and an increased rate of 111% from 1990 to 2014 [12].

The majority of injuries in young soccer players are caused by acute events resulting from player-to-player contact, especially during competitions [18,19], and by overuse, especially in the lower extremities (ankles and knees) [20,21]. It must be considered that the type and location of injuries differ by age, with 5–14 year old players more likely to be exposed to upper-extremity injuries [1,12,22] in comparison to their older peers.

Only a few studies were aimed at identifying risk factors for injuries in youth soccer, such as neuromuscular imbalances, fatigue, biomechanical factors, and previous injuries [15,21,23,24]. However, in addition to these, other factors which may increase the potential for injury in sport should be analyzed. Although there is an increasing interest in the role played by body composition, and anthropometric and growth characteristics as injury risk factors in youth soccer, the research on this topic is still scarce and controversial [21,25,26,27].

Previous studies suggested that the player’s age, evaluated as growth and maturation, is an important risk factor, especially in the life period around the peak height velocity (PHV) [25,26,28,29]. PHV is considered the moment with the largest increase in stature, implying a high growth velocity [30]. In particular, an increased likelihood of overuse injuries was observed during this adolescent growth spurt. During rapid growth, skeletally immature athletes do not have the same resistance to tensile, shear, and compressive forces as skeletally mature athletes or athletes in a prepubertal stage of greater immaturity [31]. Moreover, the anthropometric changes associated with adolescent growth spurts affect coordination skills and, consequently, the injury risk [32]. Moreover, a decrease in flexibility and bone density occurs during the maturation process, increasing the vulnerability of the skeletal system [31,33]. In a recent study, Rommers et al. [11] reported that age, total growth, and lower-limb growth were strongly associated with increased injury risk in both preadolescent and adolescent elite soccer players. According to this research, body weight and lower-limb length were also correlated with injuries [11]. From this perspective, an increased body mass index (BMI) and a low fat percentage were identified as anthropometric injury risk factors in elite-standard young soccer players [25,34]. However, the results concerning the association between body composition parameters and risk of injuries are controversial. It is possible that there could be an optimal range in fat and fat-free mass percentage in terms of injury risk, whereby every deviation could represent a potential injury risk [35,36,37]. Further studies are needed to understand the effect of growth rate, maturation, and anthropometric characteristics on the increase in injury rate risk.

Therefore, the main aims of this study were to: (i) describe the prevalence of injuries in a sample of pre-adolescent elite soccer players of different ages, and (ii) evaluate whether anthropometric characteristics, growth, and maturation are possible predictors of injury risk. We hypothesized that great changes in anthropometric characteristics and being close to the growth spurt would have a negative influence on the injury risk of young players.

## 2. Materials and Methods

### 2.1. Participants and Procedures

**Figure 1:**
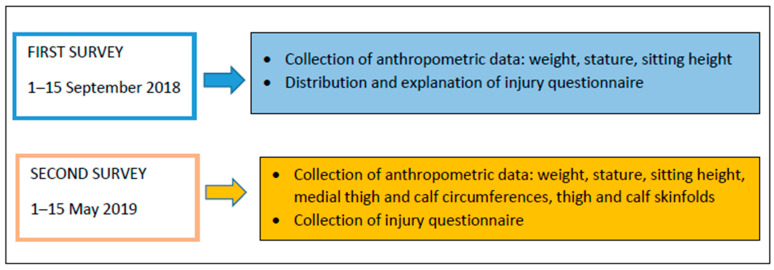
Schematic figure showing the experimental design of the study.

This research was carried out during the 2018–2019 competitive season from September to May. Figure 1 shows the experimental design of the study.

Anthropometric data were collected during the first two weeks of September in an appropriate indoor space at the soccer field. Moreover, the tracking of these anthropometric traits was investigated using a prospective design (second survey in the first two weeks of May), supplementing the anthropometric profile of soccer players with some other useful measurements for the assessment of body composition. The occurrence of injuries per season was also recorded using questionnaires distributed at the beginning of the season and collected at the end of the season. The coaches of each team were asked to check that all injuries were recorded. The injuries associated with the anthropometric characteristics and biological maturity of the players were assessed.

The study protocol was approved by the Ethical Committee of the Province of Ferrara (N. 140797). Children could only participate in the study if their parents/guardians provided informed consent. Participants were also informed that participation was voluntary and that they could withdraw from the study at any time.

This research monitored 88 male players aged 9–13. This convenience sample was recruited from teams under 13 (U13) (26 players), U12 (22 players), U11 (17 players), U10 (12 players), and U9 (11 players) of the youth academy of the professional soccer club S.P.A.L. (Società Polisportiva *Ars et Labor*, Ferrara, Italy), playing in the first division of the Italian soccer league. The sample (aged 11.5 ± 1.4 years, on average) was divided into two subgroups U9–U11 (aged 10.2 ± 0.8 years) and U12–U13 (aged 12.5 ± 0.5 years). The training program of this elite academy included at least three training sessions and one match per week, usually on Saturday. In particular, U9–U11 participants trained three days a week with a total weekly soccer training duration of 5 h and 30 min. U12–U13 participants trained four days a week with a total weekly soccer training duration of 7 h.

### 2.2. Anthropometric Measurements

At the start of the competitive season (September), we measured the following anthropometric traits: weight, stature, and sitting height. The length of the lower limb was determined using an indirect method. The repetition of these anthropometric measurements, collected during the first survey and particularly relevant for the assessment of the child’s growth, and the measurement of other anthropometric characteristics for the assessment of body composition, were included in the second survey at the end of competitive season (May): stature, weight, sitting height, lower-limb circumference (medial thigh and calf) and skinfold thickness (thigh and calf). In selecting the anthropometric characteristics, we took into account the “fundamental” anthropometric measurements (height and weight) [38] and those measurements (length of the lower limb, obtained from stature minus sitting height, and body composition parameters) considered crucial during the prepubertal and pubertal periods [39].The changes for the season in stature, weight, lower limb length, and BMI were computed and then transformed into yearly growth rates by dividing the change by the time between the two measurements. Anthropometric characteristics of children were collected according to standardized methodologies [40,41,42].

Stature and sitting height were measured to the nearest 0.1 cm using an anthropometer (Raven Equipment Ltd., UK). During the stature measurement, the child was upright without shoes, with heels together, and with the head on the horizontal plane of Frankfurt. During the sitting height measurement, the child was sitting on a box of known height with his head oriented on the Frankfurt plane. By subtracting the height of this box from the measured height, sitting height was determined. Weight was measured to the nearest 0.1 kg using a Seca weighing scale (Seca Deutschland Medical Measuring Systems and Scale, Hamburg, Germany) for children dressed in light clothing. The lower-limb length (subischial) was indirectly obtained as stature minus sitting height. Thigh and calf circumferences were measured to the nearest 0.1 cm using an anelastic tape (GPM measuring tape, DKSH, Swiss).

The same trained operator (N.R.) performed a double measurement of thigh and calf skinfold thicknesses to the nearest 0.5 mm using a Lange skinfold caliper (Beta Technology Inc., Houston, TX, USA). In the analysis, we used the average of two plyometric values. The technical error of measurement (assessed prior to project start) was <5% for skinfolds and <1% for other measurements.

All bilateral measurements were conventionally taken on the left side.

Among the anthropometric indices, we calculated the body mass index (BMI) as weight (kg)/stature squared (m^2^) and the Skelic index as (lower limb length/sitting height) × 100. Young players were classified as underweight, normal weight, overweight or obese according to international age- and sex-specific BMI thresholds [43,44]. Body composition and cross-sectional areas of the thigh and leg were also estimated from thigh or calf circumference (C) in cm, and thigh or calf skinfold thickness (ST) in cm [45,46] as follows:

Total area (cm^2^) = C^2^/4π,
Muscle area (cm^2^) = (C − (π × ST))^2^/4π,
Fat area (cm^2^) = Total area − Muscle area,
Fat Index (%) = (Fat area/Total area) × 100.

### 2.3. Peak Height Velocity

To determine the age at PHV of the players, we applied a sex-specific equation developed in Canadian and Belgian boys [30] on the basis of some anthropometric traits described above. The predictive equation [47] for boys is as follows: −9.236 + (0.0002708 × (lower-limb length × sitting height)) + (−0.001663 × (age × lower-limb length)) + (0.007216 × (age × sitting height)) + (0.02292 × (weight/stature × 100)). This equation predicts maturity offset (time before or after PHV). According to the estimated year of PHV, four maturity categories (YPHV = decimal age–PHV) were identified: category 0, YPHV >−1 year; category 1, −2 years < YPHV ≤ −1 year; category 2, −3 years < YPHV ≤ −2 years; category 3, YPHV ≤ −3 years.

### 2.4. Injuries

The injuries suffered by players during the 2018–2019 competitive season were collected through questionnaires distributed and explained at the beginning of the season. Players reported type, severity, and mechanism of injury (acute or overuse) according to the Fuller et al. [48] questionnaire. In compliance with these authors, injuries were defined as any physical complaint suffered by a player resulting from a soccer match or training, regardless of the necessity of medical care or time lost from soccer activities. Injuries resulting from a specific and identifiable event were denoted as acute, while injuries caused by repeated microtrauma without a single identifiable event were denoted as overuse. Injury number was reported separately by each player. Their severity was reported as time lost (number of days that the player was not able to take full part in competition or training): slight for 0 days; minimal for 1–3 days; mild for 4–7 days; moderate for 8–28 days; severe for >28 days [17,28,48]. Data on injury location and type were also collected. In terms of anatomic location, injuries were grouped for simplicity into three main body regions: trunk, upper limbs, and lower limbs [27].

We calculated injury incidence as the number of injuries per 1000 exposure player-hours in training and matches, as follows: (Ʃ injuries/Ʃ exposure hours) × 1000 [17,49].

### 2.5. Statistical Analysis

Descriptive statistics (means and standard deviations) were separately computed for the younger (U9–U11) and older (U12–U13) teams. We tested normality using the Kolmogorov-Smirnov test. The log-transformed values of thigh and calf skinfolds were carried out before statistical comparisons. Percentage frequency was computed for qualitative variables (weight status and YPHV categories).

Anthropometric variables were compared between injured and uninjured subgroups using the Student *t*-test for independent samples, and Cohen’s d was calculated to estimate the effect size. By convention, *t*-test effect size (ES) values of 0.2, 0.5, and 0.8 are considered small, medium, and large, respectively [50]. Differences between observed frequencies of qualitative traits (weight status; YPHV categories) and their frequencies expected under the null hypothesis were tested using the chi-squared test.

Multivariate logistic regression analysis was executed to investigate the injury risk factors. The dependent variable (selected criterion) was the injury occurrence (no injury = 0, injury = 1), while the independent predictor variables for multivariate analysis were identified using automated backward stepwise selection. The general fit of the model was tested using the Hosmer and Lemeshow goodness-of-fit test (LS). The power of the model was determined by Nagelkerke’s R-squared. The odds ratios (ORs) and 95% confidence intervals (CI) were computed referring to the injury occurrence.

Analyses were done with STATISTICA software, version 11 (StatSoft, Tulsa, OK, USA).

Statistical significance was set at *p* < 0.05.

## 3. Results

### 3.1. Anthropometric, Maturation and Injury Characteristics

In the 2018–2019 competitive season, we surveyed a sample of 88 elite soccer players. Table 1 shows the anthropometric characteristics of the total sample and of the two age subgroups (U9–U11 and U12–U13) according to the survey. As expected, the older group had higher mean values of anthropometric variables in both surveys, except for skinfold thicknesses. With regard to the weight status, there were 11 underweight (12.5%), 72 normal weight (81.8%), and five overweight (5.7%) players during the first survey (i.e., in September), whereas, at the end of the competitive season (i.e., in May), there were five underweight (5.7%), 80 normal weight (90.9%), and three overweight (3.4%) players, with non-significant changes in frequency. The growth rate in weight, stature, lower-limb length, and BMI were higher in older than in younger players; the former players were, on average, closer to maturity offset, as highlighted by the lesser distance from peak height velocity (PHV).

Table 2 displays the descriptive statistics of injuries. During the 8-month period, a total of 57 injuries were registered in the total sample: 34 injuries (equal to 60%) were a result of overuse, whereas the remaining 23 (equal to 40%) occurred traumatically. Of the injuries, 40 occurred during matches, and 17 occurred during training sessions. A total of 37 players (equal to 42% of entire sample) suffered no injury, 47 players (53.4%) suffered one injury and four players (4.5%) suffered multiple injuries. Half of the younger group (i.e., U9–U11) was injured, sustaining a total of 22 injuries (equal to 38.6% of all injuries), while 64.6% of the older group (i.e., U12–U13) was injured, sustaining a total of 35 injuries (the remaining 61.4% of all injuries). The number of injuries and the number of injured players increased with age, albeit not significantly, as highlighted by the higher injury incidence in the older group (0.73 vs. 0.55). The injury incidence for 1000 h of exposure was 2.71 in the younger and 2.82 in the older subgroup. The majority of injuries were to the lower limb (*n* = 44; 77.2%), followed by injuries to the upper limb (*n* = 7; 12.3%) and trunk (*n* = 6; 10.5%). The most frequent injuries among the players were strains, sprains, and contusions. As regards injury severity, the half (50.8%) of the injuries resulted in absence from sport fewer than 8 days, 28.1% in absence 8 to 28 days, and 21.1% in absence more than 28 days. In both age groups, there were similar proportions with regard to the type of injury (~60% for overuse and ~40% for acute); however, in the younger group, the incidence of both overuse and acute injuries was lower, especially with respect to overuse.

### 3.2. Injury Risk Factors

Table 3 shows the comparison between injured and uninjured players at the end of the competitive season. The group of injured players was significantly closer to maturity offset, with higher BMI and growth rates in stature and weight. According to the benchmarks suggested by Cohen [50], the effect size was large for BMI and rate of weight gain, whereas it was medium for rate stature gain and distance from peak height velocity. Repeating the analysis separately for overuse injuries and acute injuries, the BMI, and the growth rates in weight and lower-limb length, were found to be significantly different only when comparing overuse injured and uninjured (BMI: *p* = 0.025, ES = 0.48; weight rate: *p* = 0.025, ES = 0.46; limb length: *p* = 0.047, ES = 0.42). A further comparison of injured and uninjured players within the two age subgroups confirmed the general trend with significant differences in weight (*p* = 0.011, ES = 0.85) and BMI (*p* = 0.008, ES = 0.88) in the U9-U11 subgroup, and in weight gain (*p* = 0.032, ES = 0.71) in the U12–U13 subgroup.

The lower part of Table 3 also shows the distribution of injuries in the four categories of YPHV. Upon approaching peak height velocity, the occurrence of injuries increased from the minimum of 48% registered in category 3, i.e., the furthest from maturity offset, to the maximum of 77% in the closest category (i.e., YPHV category 0).

Table 4 displays the odds ratios of the multivariate model with 95% confidence intervals and *p*-values. Logistic regression analysis revealed that three variables (YPHV, BMI, and calf muscle area) were significant risk factors for injuries: increased YPHV with 2.6-fold increased risk of injury, increased BMI with almost twofold increased risk of injury, increased calf muscle area with 13% decrease in risk of injury. The other anthropometric variables provided no further contribution to the explanation of the model in the multivariate analysis. The Hosmer-Lemeshow statistic adequately fit the data (*p* > 0.05). Nagelkerke’s statistic indicated that the model’s explained variance was 22%.

## 4. Discussion

In this study, we analyzed the prevalence of injuries in teams of young elite soccer players by age, evaluating the main anthropometric and growth factors associated with an increased risk of injuries. Our findings demonstrate a trend in the incidence of injuries with age and biological maturity (PHV), as well as an association of some anthropometric characteristics with the risk of injuries in young elite soccer players.

A higher overall number of injuries per 1000 exposure player-hours was found in this study (2.77) than in other previous studies on soccer players (1.20 in children and 1.30 in adolescent Brazilian soccer players in [27]; 0.61 in 712-year-old Czechoslovak and Swiss players in [17]), but similar to other findings from the literature (2.81 in high-school soccer players, according to [51]). Considering the impact of different sports on the level of exposure in male adolescents, the literature shows that this rate per 1000 exposures achieves its highest values in football, hockey, and basketball, and its lowest values in track, swimming, tennis, and badminton athletes [9,51,52].

The results of this study highlighted that the number of injuries varies among different age groups (U9–U11 vs. U12–U13), regardless of the type of injury (acute or overuse). In particular, older groups reported a greater number of injured players and a greater incidence in comparison to younger players, especially with regard to overuse injuries. This could be due to the increase in training load in the older groups, as reported in the study by Vanderlei et al. [27], showing that injured adolescent players trained for longer weekly hours. Although there is no concordance in the literature regarding the relationship between athlete age and injury risk, lighter and smaller soccer players are generally deemed to be less prone to injury [11]. Furthermore, it is recognized that, as the level of competition and contact in sport increases with age, so does the risk of injury in children and adolescents [9]. In soccer, as in other sports, the peak of injuries lies consistently in the age group of older adolescents [9,53]. However, a major role could be played by the maturity status, rather than by the chronological age.

Analyzing the frequencies of injuries according to the distance from the age at PHV, we found an increasing trend of injury rate with the approach of the peak, as confirmed by the ES. This was further confirmed by the comparison between injured and uninjured players, where the mean YPHV appeared significantly lower in the first group, and by the multivariate logistic regression, where the YPHV increased the injury risk more than twofold. Previous studies have consistently underlined that maturity status influences the structural body changes of young soccer players, in addition to functional growth capacity [11,21,25,26,28,32,54].

The findings of the current study support the relevance of specific anthropometric variables from the perspective of injury prevention. BMI, calf muscle area, and growth rates in weight, stature, and lower-limb length were identified as risk factors for injuries. This finding is in line with previous studies showing that numerous anthropometric changes linked to the growth spurt of adolescence [55] led to a period of increased risk of sports injuries [56], so much so that it was suggested that earlier and later players follow different training programs [28]. Therefore, regular anthropometric measurements of players (every 3 months according to [54]) should be used to monitor their somatic maturity [57] and identify specific growth phases involving different injury risk [54].

In particular, with reference to the results of our prospective study, higher growth rates in weight and stature were found to be significant risk factors; players were more likely to be injured as the growth rates in weight and stature increased. In our study the gain rate in lower-limb length, in addition to BMI and weight gain rate, was also significantly different between overuse injured and uninjured players. These growth parameters, particularly the increase in leg length and stature [55], define the tempo of maturation and are strictly connected to the PHV, whereby the growth rate increases as the YPHV tends to zero, approaching the peak. The tendency for the risk of injury to increase with increasing maturation is probably due to the high training loads [27] that overlap with the rapid yearly growth changes. A high tempo of maturation could be one of the causes of the increase in injury rate around the PHV, as underlined by other studies performed in young soccer players [11,24,28] and in young athletes of other sports [9,27]. This could be connected to a temporary increase in skeletal fragility, which increases the rate of acute injuries [58,59], and to a temporary decline in essential motor performance during years of maximal growth caused by an imbalance between lower limb and trunk growth and muscular size and strength [55,60]. Among injuries, however, it is also well known that growth-related factors predispose players to overuse injuries [31,56], as observed in our study. The increase in weight could also be a consequence of the growth [32], but this anthropometric characteristic may be influenced by several other factors; thus, it is also important to analyze the changes in BMI among injured and uninjured players. However, the results in the literature are not always consistent. A study by Rommers et al. [11] found that an increase in leg length in younger players (U10–U12) and having long legs in older players (U13-U15) were associated with overuse injuries, while a higher weight and a slower growth rate were associated with acute injuries.

Although we found no significant difference between injured and uninjured players in body composition parameters, the relevance of calf muscle area in the occurrence of injuries emerged through multivariate logistic regression analysis: injuries decreased as the calf muscle area increased. This result underlines the importance of fat free mass in the lower extremities, which strongly contributes to strength and power performance. Players with greater muscle development in the lower limbs, due to training and/or structural characteristics, have greater dynamic stability in their sporting actions with lower risk of injury. An association of muscle weakness or alterations in muscle recruitment with injury was previously reported [61]. From this point of view, it should be noted that the stage between the ages of 12 and 15 represents a very sensitive period of neuromuscular maturation, which has to be maintained through systematic training [62].

The increase in BMI, an index commonly used as a proxy for adiposity, was another significant risk factor for injuries in our sample, as evidenced by the multivariate logistic regression analysis, as well as by the statistical comparison between injured and uninjured players. Kemper et al. [25] suggested that every change in BMI, both increases and decreases, should be monitored by coaches to reduce the risk of injuries, especially if the increase is higher than the normal growth and maturation rate. Vanderlei et al. [27], analyzing different sports, reported a significant association of weight and stature with injury risk in children over 12 years, but not in younger children. In our study, the stature did not seem to be associated with injured risk, probably because the majority of elite players examined were still far from their growth spurt (i.e., PHV), growing at an average rate of 6.1 cm/year compared to the maximum growth rate reported for soccer players of 8–9.7 cm/year [63,64]. The fact that a BMI increase of at least 0.3 kg/m^2^ per month also led to an increase in the relative risk of injury [25] is supported by our results: injured players had a yearly BMI rate that was four times higher than that of uninjured players.

The major strength of this study is its prospective research design with the repetition of some anthropometric measurements relevant to the assessment of the velocity of growth over the competitive season of elite youth soccer players. Some body composition parameters were also assessed during the second survey. All anthropometric measurements were taken directly, whereas previous studies often employed self-reported measures or were restricted to BMI values in non-elite samples, using a cross-sectional research design. Our study also has two main limitations. The first is the small size of the sample, even if it comprised only a selected group of elite soccer players. Anyway, as we analyzed an elite sample, the results of this study can provide new indications useful for future in-depth studies. The second limitation concerns the questionnaire on injury data collection [48]; despite it being the result of the Injury Consensus Group activities under the aegis of the Fédération Internationale de Football Association Medical Assessment and Research Centre (F-MARC), it was completed without medical supervision. Lastly, we could not control for every other possible injury risk factor, and we could not exclude the possibility of residual confounding. Future studies need to also consider injury risk factors for players that have already reached or surpassed their PHV, when growth rates cannot provide satisfactory explanations.

## 5. Conclusions

This study highlights the remarkable effect of biological maturity, assessed in terms of PHV and anthropometric variables, on the injury occurrence of elite young soccer players. Players who are closer to their biological maturity (YPHV) are at a higher risk of injury. On the basis of our findings, we propose that anthropometric characteristics and growth rate should be monitored because they could be connected with an increased risk of injuries in young soccer players.

With regard to the practical implications of our study, the large individual variation in adolescent growth spurts needs to be taken into consideration by coaches not only during talent identification, but also during training. Indeed, training programs must be tailored according to both the chronological age and the maturity status of the players. Moreover, some body composition and anthropometric characteristics need to be monitored as part of a prevention program in order to decrease injury risk, with particular attention paid to rapid changes in weight status during early adolescence.

## Figures and Tables

**Table 1:** Anthropometric characteristics (mean and SD) for the total sample and age subgroups of elite soccer players during the first (above) and second (below) surveys.

Variable	Total Sample (*n* = 88)		U9–U10–U11 (*n* = 40)		U12–U13 (*n* = 48)	
	Mean	SD	Mean	SD	Mean	SD
**First Survey**						
Age (years)	11.45	1.37	10.15	0.83	12.54	0.50
Weight (kg)	38.47	7.48	34.07	4.95	42.05	7.31
Stature (cm)	146.67	9.80	139.38	6.05	152.58	8.15
Sitting height (cm)	75.72	4.17	73.06	2.85	77.88	3.83
Lower-limb length (cm)	70.95	6.52	66.32	4.69	74.71	5.28
BMI (kg/m^2^)	17.75	1.95	17.50	2.02	17.95	1.88
Skelic index	93.67	6.51	90.84	6.57	95.97	5.53
YPHV (years)	−2.33	0.98	−3.21	0.53	−1.63	0.61
**Second Survey**						
Weight (kg)	41.33	8.83	35.64	4.79	46.08	8.64
Stature (cm)	150.61	10.79	142.45	6.21	157.42	8.92
Sitting height (cm)	77.02	4.86	73.79	2.97	79.72	4.48
Lower-limb length (cm)	73.59	6.86	68.66	4.68	77.70	5.57
BMI (kg/m^2^)	18.03	1.90	17.52	1.69	18.45	1.98
Skelic index	95.54	6.56	93.13	6.64	97.54	5.99
YPHV (years)	−2.18	1.09	−3.13	0.54	−1.38	0.72
Weight gain rate (kg/year)	4.40	3.90	2.43	1.43	6.00	4.50
Stature gain rate (cm/year)	6.11	3.49	4.75	3.04	7.21	3.46
BMI gain rate (kg/m^2^/year)	0.43	1.38	0.03	1.09	0.75	1.52
Lower-limb length gain rate (cm/year)	4.08	3.32	3.61	3.44	4.46	3.20
Thigh circumference (cm)	39.96	3.93	37.77	3.11	41.78	3.62
Thigh skinfold (mm)	14.32	4.35	14.35	4.52	14.29	4.25
Calf circumference (cm)	30.99	2.51	29.58	1.81	32.17	2.41
Calf skinfold (mm)	8.30	3.11	8.15	3.25	8.42	3.02
Total thigh area (cm^2^)	128.30	25.74	114.29	19.41	139.98	24.65
Thigh muscle area (cm^2^)	101.03	19.59	88.50	12.30	111.47	18.44
Thigh fat area (cm^2^)	27.28	9.93	25.79	9.73	28.51	10.02
Thigh fat index (%)	21.00	5.07	22.09	5.24	20.10	4.78
Total calf area (cm^2^)	76.95	12.66	69.90	8.66	82.83	12.53
Calf muscle area (cm^2^)	64.57	10.39	58.31	6.38	69.78	10.24
Calf fat area (cm^2^)	12.38	5.08	11.59	5.06	13.04	5.05
Calf fat index (%)	15.90	5.22	16.30	5.51	15.57	5.00

Note: U, under; YPHV, years from age at peak height velocity; BMI, body mass index.

**Table 2:** Injury characteristics for the total sample and both subgroups of elite soccer players.

All Injuries	Total Sample *n* = 88	U9–U10–U11 *n* = 40	U12–U13 *n* = 48
N (%)	57 (100)	22 (100)	35 (100)
Injured players	51 (58.0)	20 (50.0)	31 (64.6)
Incidence (*n*/player season)	0.65	0.55	0.73
Injury risk per injured athlete	1.12	1.10	1.13
**Injury severity**			
slight	9 (15.8)	4 (18.2)	5 (14.3)
minimal	10 (17.5)	4 (18.2)	6 (17.1)
mild	10 (17.5)	3 (13.6)	7 (20.0)
moderate	16 (28.1)	6 (27.3)	10 (28.6)
severe	12 (21.1)	5 (22.7)	7 (20.0)
**Overuse injuries**			
N (%)	34 (59.6)	13 (59.1)	21 (60.0)
Injured players	29 (56.9)	12 (60.0)	17 (54.8)
Incidence (*n*/player season)	0.39	0.33	0.44
Injury risk per injured athlete	1.18	1.08	1.23
**Acute injuries**			
N (%)	23 (40.4)	9 (40.9)	14 (40.0)
Injured players	22 (43.1)	8 (40)	14 (45.2)
Incidence (*n*/player season)	0.26	0.23	0.29
Injury risk per injured athlete	1.05	1.13	1.00

Note: Numbers in brackets are percentages.

**Table 3:** Comparison between injured and uninjured youth soccer players by risk factors. The anthropometric characteristics and growth rate during the second survey are presented above, whereas the distribution of injured/uninjured players according to their biological maturity (YPHV categories) is presented below.

Variable	Injured *n* = 51		Uninjured *n* = 37		*p* ^a^	Effect Size
	Mean	SD	Mean	SD		
Weight (kg)	42.89	9.20	39.18	7.92	0.0513	0.43
Stature (cm)	151.65	10.29	149.19	11.44	0.2943	0.23
Sitting height (cm)	77.67	4.81	76.13	4.86	0.1427	0.32
Lower-limb length (cm)	73.97	6.54	73.06	7.33	0.5399	0.13
BMI (kg/m^2^)	18.48	2.16	17.41	1.26	**0.0086**	0.61
Skelic index	95.28	6.59	95.89	6.58	0.6634	0.09
YPHV (years)	−1.98	1.04	−2.44	1.11	**0.0478**	0.43
Rate of weight gain (kg/year)	5.34	4.45	3.08	2.43	**0.0070**	0.63
Rate stature gain (cm/year)	6.76	3.71	5.18	2.95	**0.0368**	0.47
Rate of BMI gain (kg/m^2^/year)	0.62	1.58	0.15	1.00	0.1179	0.36
Rate of gain in lower limb length (cm/year)	4.38	3.64	3.66	2.78	0.3184	0.22
Thigh circumference (cm)	40.60	4.26	39.08	3.28	0.0723	0.40
Thigh skinfold (mm)	14.94	4.66	13.46	3.77	0.1315	0.35
Calf circumference (cm)	31.32	2.57	30.55	2.39	0.1579	0.31
Calf skinfold (mm)	8.64	3.29	7.81	2.83	0.1810	0.27
Total thigh area (cm^2^)	132.61	28.33	122.37	20.58	0.0650	0.41
Thigh muscle area (cm^2^)	103.65	21.01	97.41	17.08	0.1413	0.33
Thigh fat area (cm^2^)	28.96	11.08	24.95	7.64	0.0612	0.42
Thigh fat index (%)	21.50	5.11	20.32	4.99	0.2862	0.23
Total calf area (cm^2^)	78.57	13.12	74.72	11.81	0.1602	0.31
Calf muscle area (cm^2^)	65.51	10.29	63.27	10.53	0.3198	0.22
Calf fat area (cm^2^)	13.06	5.52	11.45	4.30	0.1442	0.33
Calf fat index (%)	16.34	5.24	15.29	5.20	0.3521	0.20
**YPHV category**	***N***	**%**	***N***	**%**	***p*^b^**	**Effect size**
0	10	77	3	23	0.0625	0.41
1	16	64	9	36		
2	15	52	14	48		
3	10	48	11	52		

Note: YPHV, years from age at peak height velocity; BMI, body mass index; category 0, YPHV > −1 year; category 1, −2 years < YPHV ≤ −1 year; category 2, −3 years < YPHV ≤ −2 years; category 3, YPHV ≤ −3 years; *p* ^a^, probability associated with the *t*-test; *p* ^b^, probability associated with the chi-squared test; bold values indicate statistical significance.

**Table 4:** Multivariate logistic regression analysis of potential injury risk factors in young soccer players.

Risk Factors	OR (95% CI)	Wald	*p*
YPHV (years)	2.633 (1.170–5.928)	5.471	0.019
BMI (kg/m^2^)	1.923 (1.225–3.018)	8.079	0.005
Calf muscular area (cm^2^)	0.874 (0.786–0.972)	6.139	0.013
Hosmer-Lemeshow	9.593		0.295
Nagelkerke R^2^	0.216		

Note: YPHV, years from age at peak height velocity; OR, odds ratio; BMI, body mass index.

## Data Availability

The data presented in this study are available on request from the authors (N.R., L.Z.). The data are not publicly available due to privacy restrictions.
